# Perspectives of Stakeholders on the Sustainability of Tuberculosis Control Programme in Ghana

**DOI:** 10.1155/2013/419385

**Published:** 2013-11-28

**Authors:** Joshua Amo-Adjei

**Affiliations:** Department of Population and Health, Faculty of Social Sciences, University of Cape Coast, Cape Coast, Ghana

## Abstract

*Objectives*. To solicit the views of some key stakeholders involved in TB control in Ghana on the sustainability of the current programme and corresponding interventions and to further discuss these views in the context of improving and/or ensuring the sustainability of existing interventions and structures. *Methods*. The study employed an interpretivist (qualitative) approach in order to obtain the “lived” experiences of personnel who are involved in TB control, either directly or indirectly. Purposive sampling was applied to select 19 respondents who provided data for the study through in-depth interviews (IDIs). The IDI data was analysed inductively in a progressive manner. Thus, respective codes were allowed to emerge from the data as opposed to deductive coding where themes are precoded. *Results*. The findings reveal two main strands of views about the sustainability of the current TB control programmes: optimism and pessimism. The optimists revealed that the integration of TB into the generalised health system, integration of TB and HIV control services, the use of internally generated funds of health facilities, and a general improvement in socioeconomic conditions of the general population could provide positive pathways to sustainability. The pessimists on the other hand noted that the existing programme was not likely to be sustainable so long as much of the operational funds were derived from external sources. Largely, the views of the pessimists were influenced by their past experiences in TB control. *Conclusions*. This paper has shown both opportunities and threats to sustainability of TB control in Ghana. The opportunities and threats could be managed positively depending on how policy actors respond to the issues raised.

## 1. Introduction

Despite the fact that tuberculosis (TB) is curable, it accounts for the highest proportion of deaths caused by infectious diseases globally [1]. In 2011, there were about 8.7 million cases and 14% of these were coinfected with HIV. Of the new cases, approximately 1.4 million died. In the last few years, however, significant progress has been made in halting and reversing the spread of TB as a response to the Millennium Development Goals (MDGs). For instance, between 2010 and 2011, new cases of TB declined by about 2.2% and compared to 1990 data, TB mortality has reduced by approximately 45%. Indeed the TB related MDG has been achieved. But notwithstanding these notable achievements, the burden of TB still remains unacceptable to the public health community [1].

The question presently engaging the attention of policy makers is how to sustain the progresses made in TB control. It is to this policy and academic relevance that this paper explores the views of stakeholders in TB control in Ghana on opportunities and threats to the sustainability of the current programme. The views of stakeholders on sustainability can help in the identification of, and focus on germane issues in, planning and utilisation of available resources as well as strategies for securing new resources. It is alleged that the TB control programme in Ghana is at the continuation stage [2]. Whereas this achievement is commendable, historical evidence has shown that laxity in sustaining efforts in TB control could lead to resurgence of old diseases. For example, the abrupt epidemic of MDR in New York City in the late 1980s has been blamed on the neglect of prior interventions, which had contributed positively to the management of TB [3, 4].

Tuberculosis is one of the diseases of public health importance in Ghana, and together with HIV, they cause about 7% of all adult deaths [5]. In the early 1990s, the Ministry of Health, Ghana, established the National TB Control Programme (NTP), following rising incidence of TB. The process of establishing a control programme was fast-forwarded when the WHO declared TB as a global health emergency in 1993. The programme was initially supported by the Danish International Development Agency (DANIDA) and later by the Government of Ghana (GoG) and subsequently, the Global Fund to Fight Malaria, HIV/AIDS, and TB.

The primacy of sustaining public health programmes is not in doubt. However, the sustainability of disease control programmes that have elements of verticalization is highly unpredictable [6]. This, according to the Shiffman [7], could be attributed to instabilities and turbulences characterising the decisions on what diseases to support. Another justification for promoting sustainability of existing interventions is that the cost of reinventing weak or dead programmes and the need to regain or maintain the trust of service users can be costly [8, 9].

The theoretical frame of this paper is situated in the sustainability sciences [10, 11]. Fiksel [10] defined sustainability as the ability of a system to adapt to a wide range of external and internal pressures yet retain functioning and performance and initiate changes through innovations in order to continuously improve performance. Because health systems perform multiplicity of functions such as governance, financing, planning, service delivery, demand generation, and evaluation [12], continuous adaptations are relevant to achieving sustainability.

Just like the diversities in health system functions, sustainability of health programmes is viewed differently. Among some of the popular views on sustainability include the ability of a system to maintain benefits [13, 14]; continuation of health programmes and institutionalisation of programmes within organisational systems [15, 16]; and community capacity to continue with programmes [17, 18]. The WHO [19] also defined sustainability as “the ability of a project to function effectively, for the foreseeable future, with high treatment coverage, integrated into available health care services, with strong community ownership using resources mobilised by the community and government” [19]. Olsen [20] on the other hand defined sustainability as “the long term ability of an organisational system to mobilise and allocate sufficient and appropriate resources (manpower, technology, information and finance) for activities that meet individual or public health needs and demands.”

In this paper, sustainability is conceptualised as the perceived and actual ability of the NTP of Ghana to continuously seek and attract resources to improve or maintain the existing levels of diagnosis and treatment of tuberculosis. Perception is included in the framing of sustainability because if the perceptions are positive, it can influence the orientation of stakeholders on how they search for and allocate new and existing resources. Stakeholders are framed as people who have vested interest in a policy and their views and participation are relevant to achieve successful programme or project implementation.

The objective of this paper was therefore to explore the views of some key actors who are engaged in TB control in Ghana on the sustainability of TB control programme within the context of international or local (country) level health policy and planning discourses. The expectation was that such views could help TB policy actors to identify and inculcate some of the emerging issues as well as exploring further for others that have the potential of shaping the sustainability of the programme and the interventions thereof.

## 2. Methods

The paper draws qualitative evidence from a larger study that investigated the policy setting for TB control in Ghana. Other substantive issues explored in the larger study have been reported elsewhere [21, 22]. In this paper, we report on perceptions of policy actors and implementers who include political leaders, technocrats of the Ghana Health Service, and senior programme officers of the NTP as well as health personnel who administer services to TB patients. These categories of respondents were selected from four out of the 10 administrative regions of the country. The research instrument was pretested in a region different from the final four for the study. The final four were selected based on the incidence of TB in the country, thus, the first four with the highest incidence. In each region selected, the district with the highest TB incidence was selected and in the districts selected the facility, which reports the highest TB cases, was also selected. The findings presented in this paper are based on 19 interviews out of the 31 conducted for the larger study.

An unstructured in-depth interview guide was employed to generate the relevant data for the paper. Key issues discussed under sustainability were on whether flow (regularity or otherwise) and quantity and quality of resources could ensure sustainability. Respondents were asked to make suggestions that are likely to contribute to sustainability. All the interviews were conducted face-to-face at the respondents' offices by the author. The duration of the interviews ranged from 30 to 60 minutes. All the interviews were tape-recorded with the verbal consent of the respondents and recordings were transcribed immediately after the interviews. The Institutional Review Board of the University of Cape Coast gave ethical approval for the study. The fieldwork was conducted between March and June 2012.

A progressive approach to qualitative analysis as suggested by Miles and Huberman [23] was applied to the data. By this approach, the data collection and analysis were undertaken concurrently. An inductive approach was taken in coding the substantive themes, based on which sustainability was identified. Further analysis of the sustainability theme revealed other subthemes based on which this paper is written. Thus, as a practical step towards the analysis of the data, the transcribed text was read repetitively. On each occasion, the respective themes that were identified were written in designated codebook. When the identified codes were considered exhaustive, they were compared to isolate distinct and similar ones. Similar themes were merged where appropriate to capture a common view while distinctive ones remained standing alone. Following this, three colleagues who are experts in qualitative data coding reviewed the coded data. Discussions were held with all the reviewers and all rough edges were smoothen. All the analyses were done manually.

## 3. Findings

The respondents had worked with the NTP of Ghana for two to 15 years at the time of data collection. At the health facilities, the respondents were mainly females and worked at district hospitals because of the inclusion criteria mentioned in Section 2. At the national, regional, and district offices, respondents were largely males. In response to the question on sustainability, two principal views emerged from the respondents. These were the optimists and pessimists, although the former appeared to be the loudest. These issues are presented in the subsequent sections.

### 3.1. Optimism about Sustainability of TB Control Programme in Ghana

Among the respondents who were optimistic about the sustainability of the programme, two main reasons were proffered to support their claims or views. First, there was a perception that because the NTP of Ghana has elements of both vertical and horizontal programme, there were prospects for TB control activities to remain a priority of the Ghana Health Service. The following extract demonstrates the typical views of such respondents.

*Luckily, integration reduces the burden that would have been associated with personnel salaries and benefits apart from those of us at the national office being paid by GF. Currently, most of our things—drugs, reagents, and laboratories materials are supplied with funds from GF but these items were being provided by the government before GF came. Therefore, we have some level of certainty that when GF winds-up, we can fall on government for assistance (National officer). *

Another closely related view to the above was the view that because TB remained on the epidemiologic profile of the country, it will continue to receive the attention of policy makers. It was the view of such respondents that TB will continue to remain a priority of GHS, especially given its positive correlation with HIV/AIDS. A respondent noted:

*I believe it is sustainable because so far as it is one of the diseases in Ghana, even in absence of Global Fund support, I think GHS would be able to continue, more especially given its positive relationship with HIV (DOTS centre nurse, public facility).*

Some other respondents expressed the view that integration into the generalised health system provides prospects for sustainability. To such respondents, there were avenues within the general system where resources could be generated for TB activities. One of those areas that were repeatedly mentioned was the use of internally generated fund (IGF). Presently, however, respondents indicated that IGFs are not used for TB activities. This was also because TB had dedicated funds from donors and they felt no need to commit additional resources into the programme. One senior technocrat surmised:

*Currently, we don't devote our internally generated funds into tuberculosis programme. It is not reasonable to do so because there is dedicated fund for TB. We can support but this may be on hold till a time when the management of the disease is completely reverted to hospitals and clinics (Technocrat, GHS). *

Other optimist indicated that sustainability should not be a prime concern of programme officials because being much concerned about sustainability had the potential of “killing” good initiative, despite it being important. The argument was that it is the degree of dependence on external resources for good initiatives that determined sustainability or otherwise. He noted:

*… Sustainability has been used wrongfully to kill several good initiatives. People throw in the question of sustainability at new initiatives and people say this is not sustainable and as a result, some good programs have not come become agenda … we should not entirely depend on external funding. It is important but not entirely. Government should be able to raise funds to support the programme (National officer). *

### 3.2. Pessimism about Sustainability of TB Control Programme in Ghana

The views of the pessimist were mainly informed by the current flow of resources for TB control programme. The arguments of the pessimists were based on their assessment of the funding characteristics of the programme. The key concern here was in relation to how the programme operated during periods when funding was provided by the Government of Ghana in contrast to when bilateral and multinational donors/institutions provided funds. The following narrations show how some of the respondents perceived sustainability based on the past and current experiences:

*When initial resources from the DANIDA were exhausted, there were cuts in resource allocation for TB. For instance, when we needed funds for TB activities, we had to write to resource managers at the GHS/MOH headquarters for approval, and the request was either denied or unduly delayed (National Officer A). *

*Let me be sincere with you, in the absence of the Global Fund's support, there will be real problems. It is not like the government is not fully committed but the problem is budgetary constraints… Now the AIDS programme is suffering because we could not secure the Round 10 grants for AIDS. Although the government promised some funds to the AIDS programme, we have not been able to redeem that promise (Member of 5th Parliament Select Committee on Health).*

For instance, there was a claim that when the initial funding provided by the Danish International Development Agency (DANIDA) was exhausted, the momentum of the NTP reduced until the Global Fund came in 2002. To some respondents also, the most appropriate long-term mechanism to achieve sustainability was to improve social interventions by targeting populations who are at high risk of infection. To those respondents, improved housing, for instance, could provide a longer-term sustainable strategy to reducing TB infections.

## 4. Discussion

The sustainability of TB control programme in Ghana was explored based on the views and experiences of certain individuals who are involved in TB control, either directly or indirectly. Regarding the question reported here, two key views were identified: optimism and pessimism and these are discussed briefly.

An important finding from this study was the view that TB control was sustainable due to the integrated approach being used to fight the disease in Ghana. There has been considerable debate about integration and verticalization of disease control programmes, particularly, in developing countries. The first model of TB control, which was based on vertical programming, is reputed to have been a failure in developing countries but successful in developed countries and the disparities in the outcomes were attributed to resource differentials [24]. The views of the respondents on integrated service being a step towards sustainability resonate with the definition of sustainable health care provided by WHO [19].

However, the Ghanaian approach to TB control is not wholly integrated. A critical analysis of the approach shows that there are some important elements of vertical approach. For instance, at the national level, the central TB unit is allowed some autonomy in financial administration, although the Ministry of Health and the Ghana Health Service provide supervisory role. This is believed to be one of the reasons for the success of TB control programme in Ghana.

We also find insights about how certain technocrats expressed “hope” due to the emerging popularity of IGF in the health system of Ghana [25], and in some hospitals, it has been reported that over 90% of funds are derived from IGF through out-of-pocket user payment and currently the health insurance [25]. The implication of relying on IGF for sustainable TB control is twofold. The first is to place TB under the health insurance scheme and this point was proposed in the NTP strategic plan of 2009–2013. The second alternative is to charge patients some fees but this may be counterproductive because most TB patients are poor and could default or abandon treatment if the cost is beyond their ability [26].

Another finding worth highlighting was the indication among respondents that working towards proper coordination and collaboration between TB and HIV services could provide impetus for sustainability. Tuberculosis is a major opportunistic infection among people living with HIV. With the advent of antiretroviral therapy, HIV patients can be expected to live longer but the risk of TB infection cannot be ignored. Given the high possibility of HIV programmes attracting more resources than TB programmes [27], this proposal appears sound. Indeed, the emerging literature on the integration of TB and HIV services supports the feasibility and cost-effectiveness of integrating services related to the two diseases [28, 29].

The position by the pessimists, which is based on “lived” experiences, pointed to a concern earlier raised by Shiffman [7]. Thus, disease control programmes, which rely heavily on external funds, could face problems of sustainability. This is because of priority setting in public health, in terms of funding and of what diseases are characterised by uncertainties [7]. Therefore, programmes that are extensively dependent on donor funding are likely to suffer when the resource is redrawn. Currently, donors provide the greater proportion of funding for TB. Even with the presence of donors, available funds since 2006 have consistently been lower than budgeted. For instance, in 2011, of the forty-four million ($44) funds that were needed for the programme in the country, only $27 million was secured, representing 60 percent budget funded. Out of the $27 million funded, Global Fund provided 64 percent while GoG provided paltry 32 percent. Available funding for 2012 is estimated at $24 million dollars, indicating a shortfall of 11 percent. Out of the $24 million available, the GF is providing about 54% ($12,960,000) while GoG is providing the remaining 46 percent (11,040,000) [30]. The need for longer-term planning and analysis of programmes which depend on donor start-up funds is critical for sustainability, particularly if the host government will be expected to continue providing funds when donors exit [31].

In spite of these gaps, some stakeholders were of the view that unbridled concerns about sustainability could cripple good initiatives, arguing that it is rather the efficient utilization of available resources which should be of concern to policy makers. Thus, if resources were applied to the purposes for which they were acquired, it would help policy makers attract additional resources to continue interventions up to the point where programmes become self-sustaining. Strict accountability principles are therefore required as part of the strategies for promoting sustainability. This is a view shared by a number of institutions that support public health programmes, either materially or financially [32].

From policy perspective, two broad approaches could be targeted. These are the biomedical approach of removing existing infections and preventing new infections through both biomedical and social interventions. In respect of the former, the national health insurance in the country provides an alternative source of funding some aspects of TB control. For instance, the scheme management could be urged to include TB diagnosis and treatment into the list of services provided by facilities registered with the scheme and funded through taxing mining and cigaret companies which provide risky environments and conditions that can facilitate TB infections and transmission [33, 34]. In the long-term, it is important that efforts are made to improve socioeconomic conditions of the poor who are at greater risk of contracting TB. Interventions such as these could have high potentials for managing the programme sustainable in both short- and long-terms.

Despite the modest contribution this exploratory study makes to our understanding of how TB control can be sustainable in a developing country setting, there are some limitations to the study. The findings presented here are not representative of all the stakeholders involved in TB control and the views reported here cannot be exhaustive of the generality of views on sustainable TB control in the country.

## 5. Conclusion

In infectious disease control, complacency can derail benefits already achieved and that portends the need for devising mechanisms that will promote sustainability. This paper has shown both opportunities and threats to sustainability of TB control in Ghana. Among the opportunities are integration of TB control into, first, the generalised health system and, second, integration of TB and HIV services as well as resorting to the utilisation of IGF and including TB diagnosis into the NHIS treatment and drugs list. Although the steps recommended are context specific (Ghana), they can be applied to TB control in other developing country settings.
